# Physiotherapy enhances gait stability in a forelimb-amputated dog undergoing postoperative radiotherapy: a case report

**DOI:** 10.1186/s12917-025-05075-2

**Published:** 2025-10-21

**Authors:** Kazuyuki Yoshikawa, Harumi Sawada, Atsushi Fujita, Eri Fukazawa, Kazuya Edamura, Akio Shimada, Tsuyoshi Kadosawa

**Affiliations:** 1 Japan Small Animal Medical Center, 1-10-4 Higashi-tokorozawa, 8, Tokorozawa, Saitama, 359-0025 Japan; 2 Laboratory of Veterinary Surgery, Department of Veterinary Medicine, College of Bioresource and Sciences, Nihon University, Fujisawa, Kanagawa 252-0880 Japan; 3 Toyo sogu, 2-26-40 Negishi, Machida, Tokyo, 194-0038 Japan

**Keywords:** Canine, Forelimb amputation, Gait kinematics, Physiotherapy, Radiotherapy, Functional recovery

## Abstract

**Background:**

Limb amputation is a standard surgical procedure in dogs for the management of limb tumors such as osteosarcoma, soft tissue sarcoma, and malignant peripheral nerve sheath tumors. While many dogs adapt functionally to limb loss, altered biomechanics, compensatory strain on remaining limbs, and impaired mobility can negatively impact their quality of life. Physiotherapy is recommended to facilitate post-amputation recovery; however, objective data on its effectiveness in dogs remain limited. Furthermore, when radiotherapy is required postoperatively, the necessary daily anaesthesia and cage rest can exacerbate functional decline due to restricted activity. The impact of physiotherapy on gait function in a forelimb-amputee dog undergoing concurrent radiotherapy is described in this report.

**Case presentation:**

A 14-year-old neutered male Toy Manchester Terrier underwent left forelimb amputation following the diagnosis of a malignant peripheral nerve sheath tumor at the C6–T1 spinal level. One month postoperatively, the dog began a three-week course of radiotherapy that required daily anaesthesia and prolonged cage rest, raising concerns about mobility deterioration. To mitigate these effects, a structured physiotherapy program was implemented. The program consisted of daily 30-minute sessions focusing on range of motion exercises, balance and proprioception training, weave pole exercises, and cavaletti rail walking. Gait analysis was performed on the first and last days of the physiotherapy program (pre- and post-physiotherapy) using a two-dimensional kinematic system.

After three weeks of physiotherapy, a reduction in vertical head movement was observed (48.3 cm to 34.5 cm), indicating improved gait stability. Additionally, shoulder and elbow extension showed an increase during the loading response phase, which is crucial for weight-bearing and locomotor efficiency.

**Conclusions:**

This case provides objective evidence supporting the potential benefits of physiotherapy in enhancing gait function and stability in dogs following a forelimb amputation, even when physical activity is restricted due to radiotherapy-related hospitalization. The findings suggest that integrating physiotherapy into post-amputation care may mitigate functional decline associated with prolonged cage rest and optimize recovery. Further studies are needed to investigate the long-term benefits of physiotherapy and effects of physiotherapy on compensatory musculoskeletal adaptations in amputee dogs.

## Background

Limb amputation is a standard surgical procedure in dogs for treating malignant limb tumors including osteosarcoma, soft tissue sarcoma, and malignant peripheral nerve sheath tumor (MPNST) [1–3]. Although dogs often adapt to limb loss, optimizing post-operative functional recovery while minimizing compensatory strain on the remaining limbs remains a significant clinical challenge.

Multiple studies have shown positive owner satisfaction when limb amputation is performed in canine patients [1, 2, 4]. However, it was also found in those studies that owners observe negative changes in their dogs’ mobility, behavior, and quality of life. Furthermore, kinetic and kinematic analyses have revealed that forelimb amputees experience substantial alterations in gait patterns and postural biomechanics, often resulting in asymmetric weight distribution and increased mechanical load on the contralateral limb [5, 6].

Physiotherapy has been proposed as a potential modality to address these challenges [7], but objective data supporting its efficacy in canine forelimb amputees are scarce. Kinematic gait analysis provides precise information for quantitative assessments of locomotion, enabling objective evaluation of rehabilitation interventions [8].

The purpose of this case report is to document the effectiveness of a three-week structured physiotherapy program, conducted on weekdays, in a dog that underwent left forelimb amputation and concurrent radiotherapy. Given that daily anaesthesia and prolonged cage rest during radiotherapy posed a risk of functional decline due to restricted mobility, physiotherapy was initiated to mitigate these effects and enhance post-amputation gait function. The impact of physiotherapy was objectively evaluated using two-dimensional kinematic gait analysis for assessing changes in gait stability and joint kinematics.

## Case presentation

A 14-year-old neutered male Toy Manchester Terrier presented to his primary care veterinarian with intermittent non-weight bearing of the left forelimb. Magnetic resonance imaging (MRI) was performed and it revealed enlargement of the left eighth cervical spinal nerve. For further evaluation, the dog was referred to our hospital, where a spinal tumor at the left C6-T1 levels was diagnosed. Considering the tumor’s location and progression, left forelimb amputation was performed. The surgery proceeded without intraoperative complications, and histopathological examination confirmed a malignant peripheral nerve sheath tumor (MPNST).

**Fig. 1:**
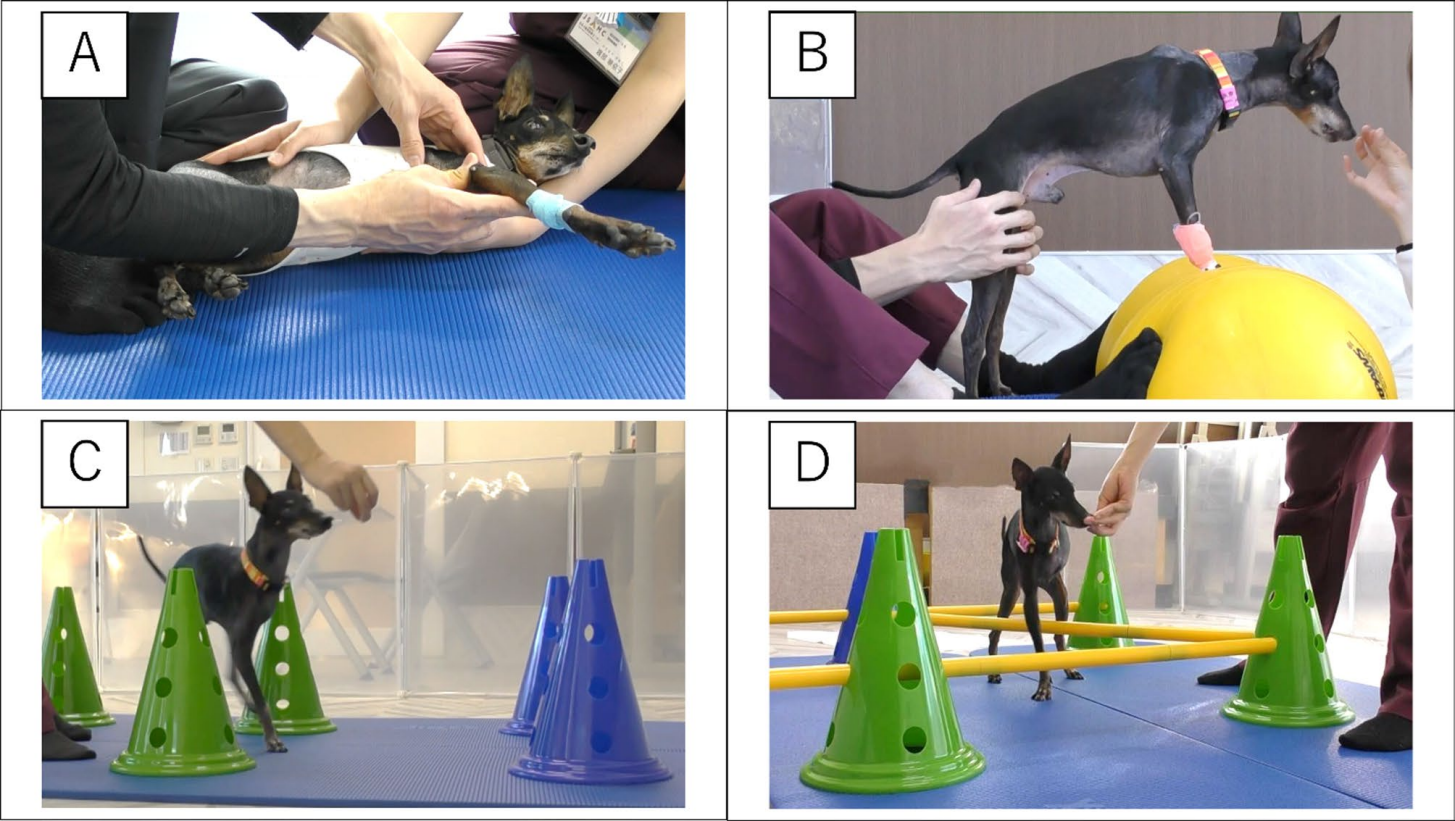
Key components of the physiotherapy program: (**A**) Range of Motion Exercises, (**B**) Balance and Proprioception Training, (**C**) Weave Pole Exercises, and (**D**) Cavaletti Rail Walking

One month after surgery, to address potential residual tumor cells, a three-week course of radiotherapy was administered on weekdays. For each radiotherapy session, general anesthesia was induced via intravenous administration of propofol (Propofol Intravenous Injection 1%; Maruishi Pharmaceutical Co., Ltd., Japan) at a dose of 5–9 mg/kg body weight, followed by endotracheal intubation. Anesthesia was subsequently maintained with sevoflurane inhalation in an oxygen–air mixture, using an animal ventilator (Spiritus, ACOMA Medical Industry Co., Ltd., Japan). The depth of anesthesia was continuously adjusted as needed to ensure immobility and patient safety. No anesthesia-related complications were observed throughout the treatment period. As the dog was confined to cage rest during this period, concerns were raised about functional decline due to decreased physical activity. To prevent this decline and promote further functional improvement, a physiotherapy program was initiated (Fig. 1).

Physiotherapy sessions were conducted once daily for 30 min with focus on the following interventions. The detailed contents of each exercise, including repetitions and duration, are summarized in Table 1. The number of repetitions or sets was moderately adjusted depending on the dog’s condition and fatigue on a given day, and short rest breaks (approximately 1–3 min) were provided between exercises as needed.

**Table 1:** Physiotherapy program with exercise details and repetitions/duration

Exercise	Details/Target	Repetitions/Duration
A) Range of Motion (ROM) Exercises	Passive ROM exercises were performed on the remaining forelimb and both hind limbs to maintain joint flexibility and prevent contractures.	~ 30 reps per limb
B) Balance and Proprioception Training	Static and dynamic exercises on balance discs and wobble boards were performed to improve proprioception and reduce compensatory weight shifting, specifically addressing the tendency observed in this case to flex the hind limbs and shift the center of mass caudally as a compensatory posture.	~ 2 min × 3–5 sets
C) Weave Pole Exercises	This exercise involved consciously incorporating irregular walking patterns by having the dog walk through poles arranged in a zigzag pattern, which helped to improve balance and enhance coordination.	2–3 reps × 3–5 sets
D) Cavaletti Rail Walking	This exercise involved stepping over obstacles to improve limb awareness and proprioception, promoting greater joint range of motion and strengthening the remaining limbs by encouraging active flexion and extension movements. The rail height was set at 7 cm (just above the carpus) during the first 1.5 weeks and increased to 14 cm (just below the elbow) from week 1.5 onward.	2–3 reps × 3–5 sets

## Recording methods

**Fig. 2:**
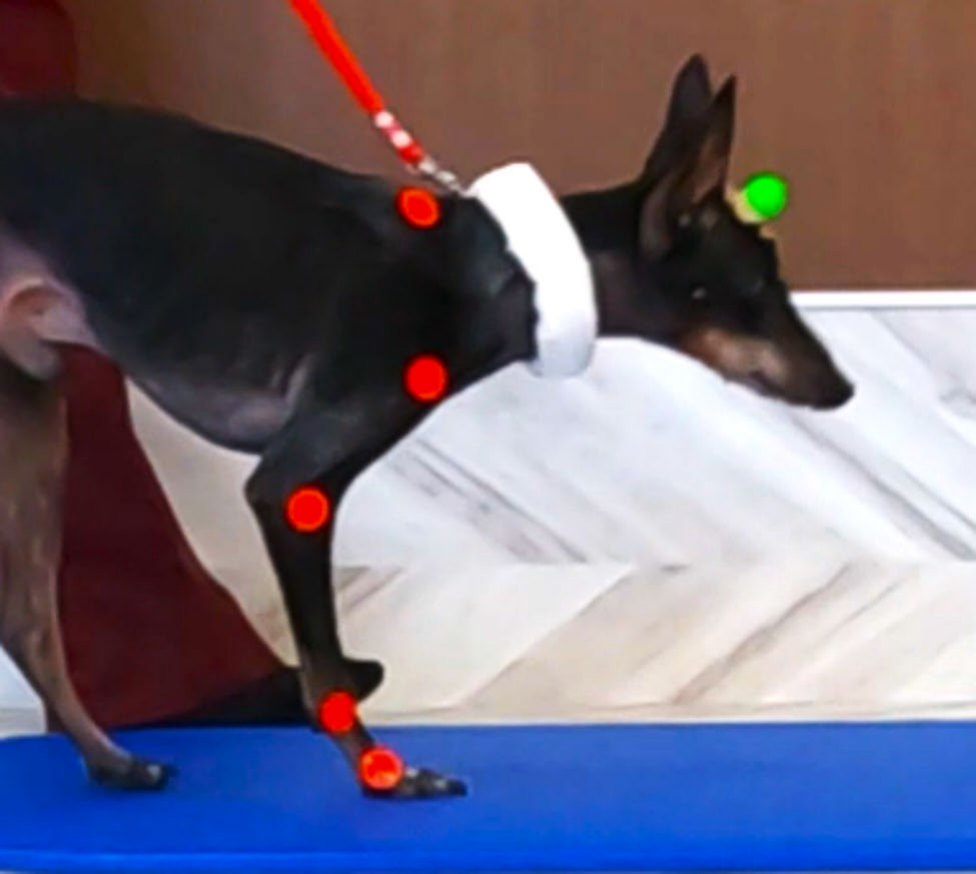
Placement of colored markers on anatomical landmarks including the midline point of the frontal bone region (forehead, dorsal to the eyes), the dorsal end of the scapular spine, the greater tubercle of the humerus, the lateral epicondyle of the humerus, the styloid process of the ulna, and the head of the fifth metacarpal bone

Video recordings focusing on the sagittal plane were obtained during overground walking at a comfortable speed for the dog. Recordings were conducted in the dedicated rehabilitation room, which provided a quiet environment free of other animals and sufficient space for straight-line walking. The walkway was prepared by connecting two exercise mats (Coronella, AIREX, Switzerland) to create an approximately 4-m overground path, allowing consistent and repeatable locomotion. To facilitate straight walking without pulling the dog, the handler practiced leash-walking alongside the dog, thereby ensuring natural and steady progression. For animal welfare, short rest periods of approximately 1–2 min were provided between trials. After the dog had become habituated to walking straight on the mat, 10 valid gait cycles were acquired at each time point (pre- and post-physiotherapy) for analysis. The gait cycle was defined as the period from the right forelimb making contact with the floor until the next contact of the right forelimb. Forelimb joint angles and head movements were assessed using a two-dimensional motion analysis system (ICpro-2D, Hutech Co., Ltd, Tokyo, Japan, 60 Hz). Color markers were attached with double-sided adhesive tape to anatomical landmarks of the forelimb and head (Fig. 2). The marker used to evaluate head movement was placed on the frontal bone region and applied as a spherical marker (green), which allowed stable attachment and ensured clear visibility during motion capture. For evaluating the range of motion of other joints, flat adhesive markers (red) were used. The anatomical landmarks included the midline point of the frontal bone region (forehead, dorsal to the eyes), the dorsal end of the scapular spine, the greater tubercle of the humerus, the lateral epicondyle of the humerus, the styloid process of the ulna, and the head of the fifth metacarpal bone.

Forelimb joint angles were calculated by defining the angles between adjacent segments. All video images were analyzed frame by frame by a single observer. Each joint angle obtained during a gait cycle was normalized to 100 time frames [9]. After normalization, the joint angles were averaged across cycles. The maximum extension and flexion angles of the shoulder, elbow, and carpal joints, as well as the vertical head movement—measured as the peak-to-peak vertical displacement of the head during gait cycles— were summarized using descriptive statistics (mean ± standard deviation, SD). A total of 10 valid gait cycles were analyzed for both pre- and post-physiotherapy conditions. No inferential statistical tests were performed, in accordance with the single-subject design of this case report.

## Outcome

**Fig. 3:**
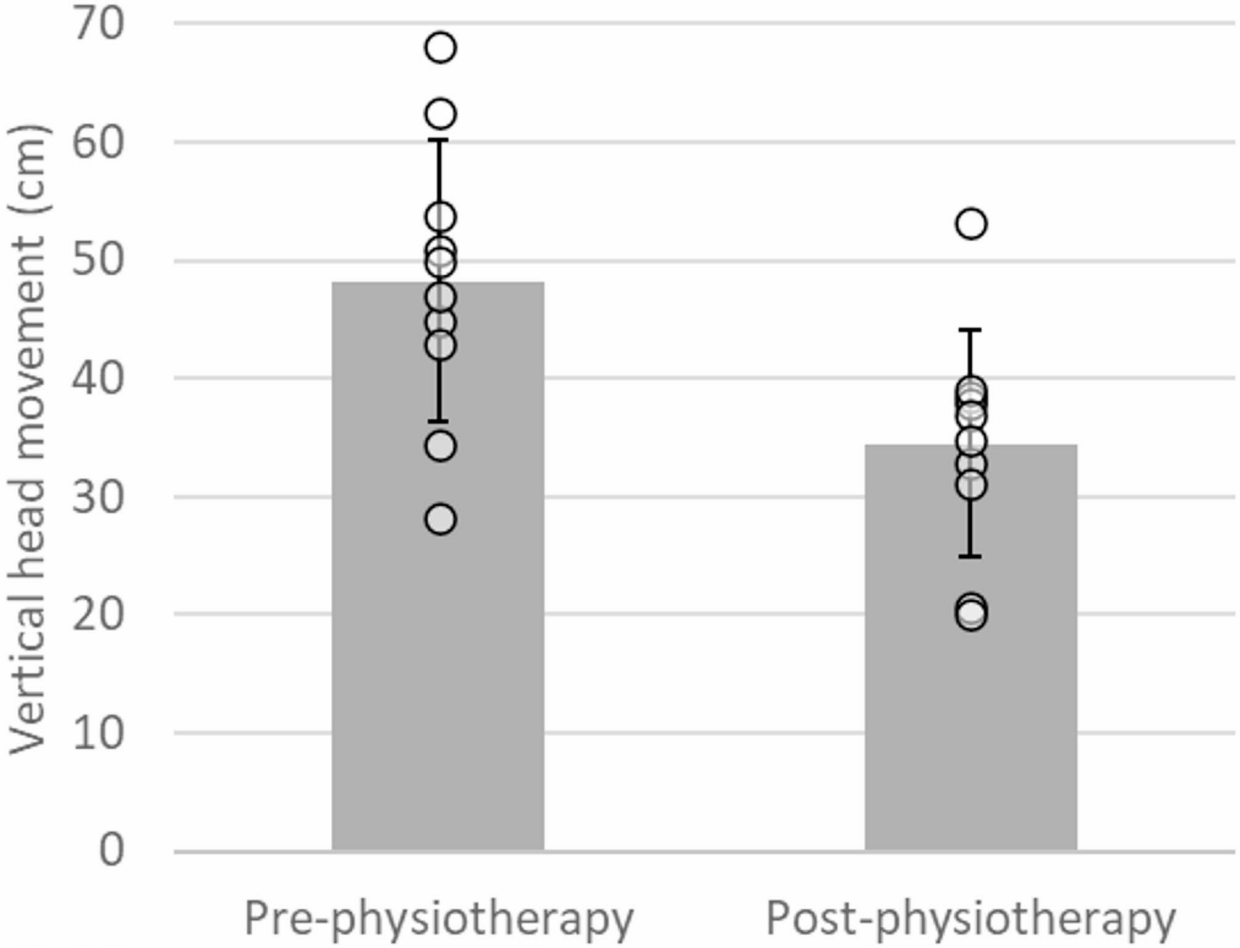
Vertical head movement pre- and post-physiotherapy. Bars indicate the mean values with standard deviation (SD). Each dot represents an individual measurement derived from 10 valid gait cycles per condition. The mean walking speed was 1.18 m/s pre-physiotherapy and 1.12 m/s post-physiotherapy

A reduction in vertical head movement was observed post-physiotherapy, with the mean value decreasing from 48.3 ± 11.9 cm pre-physiotherapy to 34.5 cm ± 9.5 cm post-physiotherapy (Fig. 3). The mean walking speed was 1.18 m/s pre-physiotherapy and 1.12 m/s post-physiotherapy, both of which fall within the reported range of comfortable walking speeds for dogs [10].

Values are presented as means ± standard deviation (SD), calculated from 10 valid gait cycles per condition. Joint angles are expressed in degrees (°). Maximum extension and flexion angles of the forelimb joints are shown for both pre- and post-physiotherapy conditions.

For joint angles, differences were observed between pre- and post-physiotherapy conditions (Table 2). In the shoulder joint, the maximum extension increased from 133.2° (± 4.7°) to 152.2° (± 4.8°) and the maximum flexion angle also increased from 101.1° (± 3.0°) to 125.5° (± 4.6°), indicating a reduction in flexion range. In the elbow joint, the maximum extension angle improved from 142.3° (± 8.6°) to 154.3° (± 6.0°) and the maximum flexion angle increased from 95.6° (± 15.2°) to 104.0° (± 5.8°), also suggesting decreased flexion. In the carpal joint, the maximum flexion angle increased from 92.0° (± 11.3°) to 111.5° (± 8.4°), reflecting reduced flexion. Although there was an increase in the maximum extension angle from 199.9° (± 8.0°) to 207.0° (± 9.3°), this change was not pronounced.

**Table 2:** Maximum extension and flexion angles of forelimb joints pre- and post-physiotherapy

		pre-physiotherapy	post-physiotherapy
Shoulder joint	Maximum Extension	133.2 ± 4.7	152.2 ± 4.8
	Maximum Flexion	101.1 ± 3.0	125.5 ± 4.6
Elbow joint	Maximum Extension	142.3 ± 8.6	154.3 ± 6.0
	Maximum Flexion	95.6 ± 15.2	104.0 ± 5.8
Carpal joint	Maximum Extension	199.9 ± 8.0	207.0 ± 9.3
	Maximum Flexion	92.0 ± 11.3	111.5 ± 8.4

**Fig. 4:**
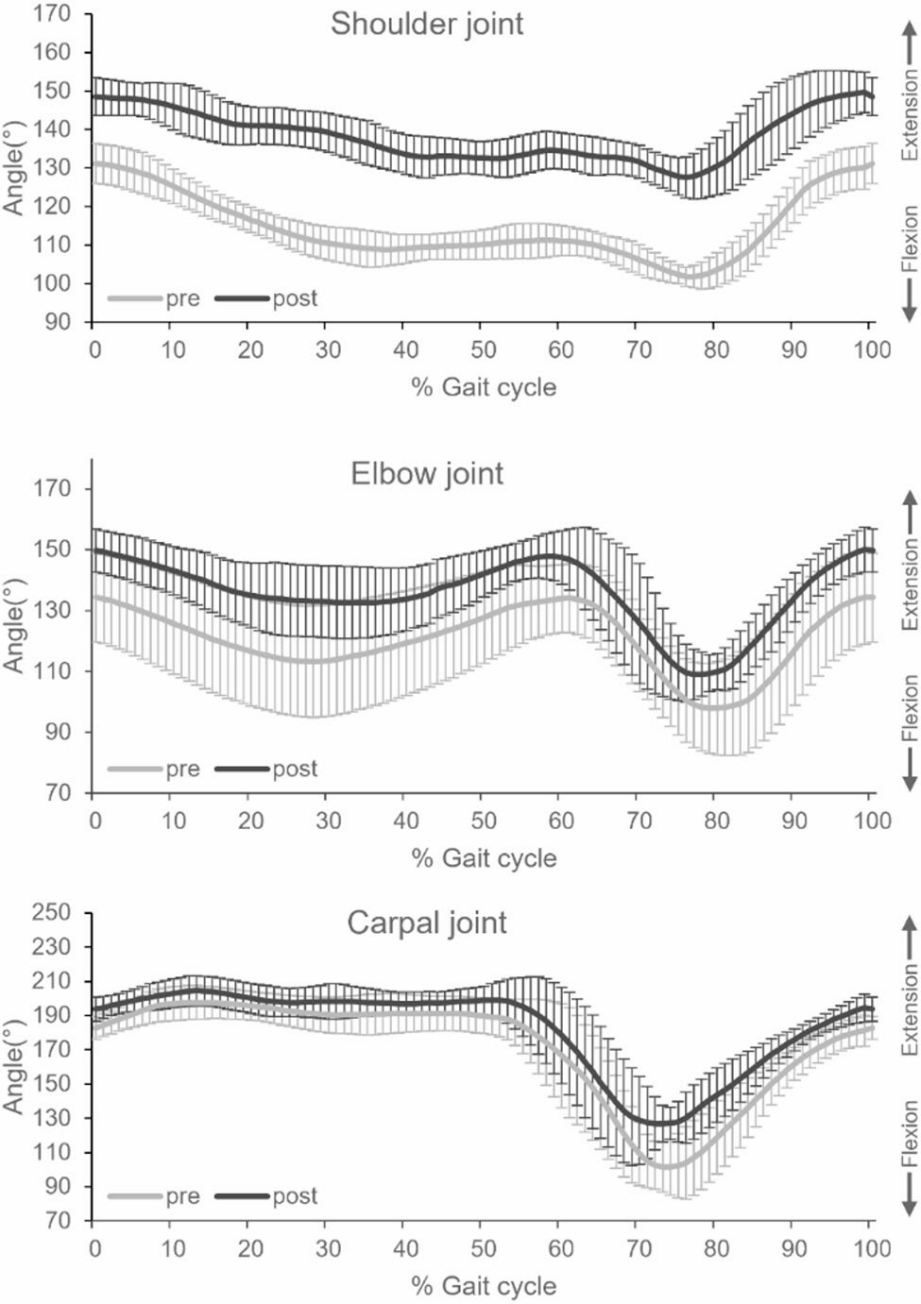
Temporal changes in shoulder, elbow, and tarsal joint angles throughout the gait cycle pre- and post-physiotherapy. Each plot represents the mean (solid line) ± SD. The gray line represents pre-physiotherapy (pre) conditions, and the black line represents post-physiotherapy (post) conditions. The x-axis indicates the percentage of the gait cycle, where 0% corresponds to the initial contact of the right forelimb and 100% represents the subsequent contact of the same forelimb. The y-axis represents joint angles in degrees, illustrating variations over time during gait progression. Changes in joint kinematics following physiotherapy intervention highlight alterations in range of motion and temporal coordination across the gait cycle

In the shoulder joint, the post-physiotherapy condition demonstrated improved maintenance of an extended position during gait compared to the pre-physiotherapy condition, as shown in Fig. 4. Additionally, in both the shoulder and elbow joints, the early phase of the gait cycle—immediately after forelimb contact—exhibited a more gradual flexion in the post-physiotherapy condition, whereas a more rapid flexion was observed pre-physiotherapy (Fig. 4). In the latter half of the gait cycle, corresponding to the swing phase, flexion decreased across all forelimb joints in the post-physiotherapy condition (Fig. 4).

## Discussion and conclusions

There were two clinically relevant findings in this case report: physiotherapy can improve gait in dogs after a forelimb amputation and combining radiotherapy with physiotherapy is beneficial.

Firstly, while physiotherapy is recommended as part of the management for amputee cases, there is a paucity of objective data demonstrating its effectiveness in dogs following a forelimb amputation. In this case, clinical improvements were noted after the physiotherapy intervention. There was a clear reduction in vertical head movement during walking, indicating enhanced gait stability (Fig. 3). The walking speed also decreased modestly from 1.18 m/s to 1.12 m/s, which may partly influence kinematic outcomes; however, both values remained within the reported comfortable walking speed range for dogs [10], and previous studies suggest that moderate speed variations of this magnitude alone are unlikely to explain such a marked reduction in head excursion. Therefore, while acknowledging the potential contribution of speed, we interpret the observed change as a descriptive trend reflecting smoother forelimb loading and improved trunk stabilization following physiotherapy. Additionally, there was increased maintenance of extension in the shoulder and elbow joints during the loading response phase, which is crucial as this phase corresponds to the peak vertical ground reaction forces [11] (Fig. 4). Furthermore, the maximum flexion angles of the shoulder, elbow, and carpal joints during the swing phase were reduced (Fig. 4; Table 2), indicating that the limb remained in a relatively more extended position at peak flexion. Although no statistical analysis was performed, these mean value changes suggest a trend toward smoother limb trajectories following physiotherapy. Excessive flexion of the forelimb during the swing phase, often observed as a compensatory adaptation in amputee or lame dogs, has been reported to increase energetic cost and trunk oscillations, thereby compromising gait stability [12, 13]. In this case, the observed reduction in swing-phase flexion following physiotherapy likely reflects improved neuromuscular control and more efficient limb trajectories, facilitating smoother limb advancement and reducing trunk instability. Together with increased shoulder and elbow extension during stance, and normalized carpal flexion during swing, these changes contributed to enhanced stability and gait efficiency, consistent with previous reports [5, 7, 14]. These observed trends suggest that physiotherapy can effectively enhance gait patterns in canine forelimb amputees. To our knowledge, this is the first report to provide objective, clinically meaningful evidence of the effectiveness of physiotherapy in such cases.

In dogs, vertical head movement is closely coupled to forelimb loading: when a forelimb is unloaded or painful, the head typically rises during stance of that limb and drops during stance of the contralateral “sound” limb, increasing peak-to-peak excursion and asymmetry of head motion. This pattern has been quantified with inertial/kinematic approaches in canine lameness models and is widely used clinically to interpret forelimb loading [12, 13]. In the context of forelimb amputation, the remaining forelimb must absorb greater vertical impulse and altered loading patterns. This increased demand places substantial strain on the forelimb extensor muscles and the pectoral muscular sling. When these structures cannot fully compensate, excessive trunk and head oscillations may occur, predisposing to gait instability and increasing the risk of overuse of the remaining limbs [15]. Accordingly, the reduction in vertical head movement we observed post-physiotherapy likely reflects smoother, more consistent forelimb loading and improved trunk stabilization (Fig. 3). Although no statistical inference was performed in this single-case design, the observed reduction provides clinically meaningful evidence of enhanced gait stability.

Secondly, the combination of radiotherapy and physiotherapy appeared to provide clinical benefit. During the three-week course of radiotherapy, the dog underwent daily anaesthesia and was confined to cage rest, resulting in markedly reduced activity levels compared to home life. Despite these limitations, implementing physiotherapy during hospitalization led to notable improvements in gait function. While physiotherapy is commonly employed after conditions like intervertebral disc herniation or orthopedic surgeries, this case demonstrates that even when radiotherapy is the primary treatment modality, adjunct physiotherapy can support gait quality and overall well-being.

Specifically, the physiotherapy interventions implemented likely contributed to functional improvements through distinct biomechanical mechanisms (Table 1). A) Range of motion (ROM) exercises helped to maintain and possibly enhance joint flexibility and prevent contractures, which are essential for fluid limb movements and compensatory gait patterns post-amputation [7, 16]. B) Balance and proprioception training using balance discs and wobble boards potentially improved neuromuscular control and dynamic stability by enhancing sensory feedback mechanisms, reducing compensatory weight shifting, and thereby challenging neuromuscular control of forelimb weight acceptance and trunk stabilization under variable support conditions. These exercises are widely employed in canine rehabilitation to promote postural stability and sensorimotor integration, and their relevance is heightened in amputee dogs where the remaining forelimb–trunk support system must absorb increased load [7, 16, 17]. C) Weave pole exercises involved consciously incorporating irregular walking patterns by having the dog walk through poles arranged in a zigzag pattern, promoting controlled lateral weight shifts, precise foot placement, and coordinated turning. Such directional-change tasks are recognized to increase forelimb muscular demand and trunk engagement in canine rehabilitation, providing functional carryover to everyday turning and maneuvering [7, 18]. (D) Cavaletti rail walking involved stepping over obstacles to improve limb awareness and proprioception, encouraging physiologic stride length and controlled sagittal-plane motion while reinforcing stable forelimb load acceptance and trunk steadiness during repeated “mini‑perturbations.” Cavaletti work is widely recommended to refine joint excursion and neuromuscular control in canine rehabilitation, and its rationale aligns with the heightened trunk‑support requirements of tripedal gait [7, 14, 17]. Improved neuromuscular control across these combined modalities likely contributed to the observed reduction in vertical head movement by facilitating stable forelimb extension during stance and smoother swing-phase trajectories, ultimately enhancing overall gait stability. In this case, neuromuscular electrical stimulation (NMES) was not applied because the dog had limited tolerance for prolonged restraint and immobility, which are required for the safe and effective administration of NMES. However, previous studies have suggested that combining NMES with conventional physiotherapy exercises can further enhance muscle strength and functional recovery [19, 20]. It is therefore possible that, had NMES been tolerated and implemented, additional improvements in muscle strength of the remaining three limbs and overall gait function might have been achieved.

Previous studies, such as the study by Kirpensteijn et al., suggested that the adaptation period after amputation is approximately one month [1]. In this case, physiotherapy was initiated about one month post-amputation. At the onset of physiotherapy, the dog was able to walk without falling; however, progressive improvements in gait were observed following the three-week physiotherapy intervention. Other reports of physiotherapy in canine amputees are limited, but in related conditions such as intervertebral disc herniation or orthopedic surgery, rehabilitation is often initiated within days post-treatment, and earlier initiation has been associated with improved outcomes [7, 16, 21]. This suggests that while physiotherapy for amputees may sometimes be delayed due to surgical or oncological considerations, variability exists in adaptation periods among individuals, and meaningful gait improvements can still be achieved even when initiated after the initial adaptation period. In addition, locomotor treadmill training was not performed in this case because the dog had difficulty habituating to the specific sounds and mechanical stimuli of the treadmill. However, treadmill-based gait training has been reported to facilitate locomotor recovery and improve endurance and coordination in canine rehabilitation [22, 23]. Therefore, although not feasible in this individual, treadmill training could represent a valuable adjunct in suitable patients and should be considered as part of a comprehensive rehabilitation strategy. Moreover, incorporating physiotherapy—even when it is not the primary treatment focus—can contribute to better motor function and improved quality of life in canine patients.

These findings highlight the potential of physiotherapy to enhance gait function in dogs following a forelimb amputation. Additionally, the integration of physiotherapy with radiotherapy may help to mitigate mobility decline associated with prolonged hospitalization and restricted activity. Even when dogs regain the ability to walk after amputation, physiotherapy can further refine gait quality and stability (Figs. 3 and 4; Table 2). Moreover, implementing physiotherapy concurrently with specialized treatments such as radiotherapy may optimize overall treatment outcomes. However, the relatively short duration of the present study limits the conclusions that can be drawn regarding the long-term effects of physiotherapy on gait adaptation and musculoskeletal health. Furthermore, as only two-dimensional kinematic and vertical head movement analyses were employed, the full complexity of compensatory gait mechanisms may not have been captured. In addition, because this study describes a single case, the findings are limited in their generalizability to the wider population of canine amputees. Rather, this report should be regarded as preliminary evidence highlighting the feasibility and potential clinical utility of physiotherapy in this context, thereby providing a rationale and foundation for future controlled studies with larger cohorts. Future research should therefore extend the follow-up period to assess longer-term outcomes, and incorporate three-dimensional kinematic measurements alongside ground reaction forces to provide a more comprehensive understanding of locomotor adaptations. Additionally, further investigations are warranted to explore the long-term benefits of physiotherapy in amputee dogs, assess its role in preventing compensatory musculoskeletal strain, and evaluate its effectiveness when combined with other therapeutic modalities.

## Data Availability

All data generated or analyzed in this study are presented within this published article.

## Abbreviations

MPNST: Malignant peripheral nerve sheath tumor
MRI: Magnetic resonance imaging
NMES: Neuromuscular electrical stimulation
ROM: Range of motion

## References

[1] Kirpensteijn J, Van den Bos R, Endenburg N. Adaptation of dogs to the amputation of a limb and their owners’ satisfaction with the procedure. Vet Rec. 1999;144(5):115–8. 10070700 10.1136/vr.144.5.115

[2] Wendland TM, Seguin B, Duerr FM. Prospective evaluation of canine partial limb amputation with socket prostheses. Vet Med Sci. 2023;9(4):1521–33. 37287388 10.1002/vms3.1146 PMC10357256

[3] Withrow SJ, Hirsch VM. Owner response to amputation of a pet’s leg. Vet Med Small Anim Clin. 1979;74(3):332. 255302

[4] Dickerson VM, Coleman KD, Ogawa M, Saba CF, Cornell KK, Radlinsky MG, Schmiedt CW. Outcomes of dogs undergoing limb amputation, owner satisfaction with limb amputation procedures, and owner perceptions regarding postsurgical adaptation: 64 cases (2005–2012). J Am Vet Med Assoc. 2015;247(7):786–92. 26383755 10.2460/javma.247.7.786

[5] Jarvis SL, Worley DR, Hogy SM, Hill AE, Haussler KK, Reiser RF. Kinematic and kinetic analysis of dogs during trotting after amputation of a thoracic limb. Am J Vet Res. 2013;74(9):1155–63. 23977887 10.2460/ajvr.74.9.1155

[6] Cole GL, Millis D. The effect of limb amputation on standing weight distribution in the remaining three limbs in dogs. Vet Comp Orthop Traumatol. 2017;30(01):59–61. 27977027 10.3415/VCOT-16-05-0075

[7] Millis D, Levine D. Canine rehabilitation and physical therapy 2edn. USA: Elsevier Health Sciences; 2014.

[8] Gillette RL, Angle TC. Recent developments in canine locomotor analysis: a review. Vet J. 2008;178(2):165–76. 18406641 10.1016/j.tvjl.2008.01.009

[9] Yoshikawa K, Tsubakishita S, Sano T, Ino T, Miyasaka T, Kitazawa T. Functional assessment of the gluteus medius, cranial part of the biceps femoris, and vastus lateralis in Beagle dogs based on a novel gait phase classification. J Vet Med Sci. 2021;83(1):116–124. 10.1292/jvms.20-0127 PMC7870396 33229819

[10] Blake CA, Looney AL, Merrill TD. The impact of Cavaletti height on dogs’ walking speed and its implications for ground reaction forces. Front Veterinary Sci. 2024;11:1419206. 10.3389/fvets.2024.1419206 PMC11301751 39109348

[11] Stark H, Fischer MS, Hunt A, Young F, Quinn R, Andrada E. A three-dimensional musculoskeletal model of the dog. Sci Rep. 2021;11(1):11335. 34059703 10.1038/s41598-021-90058-0 PMC8166944

[12] Rhodin M, Bergh A, Gustås P, Gómez Álvarez CB. Inertial sensor-based system for lameness detection in trotting dogs with induced lameness. Vet J. 2017;222:54–9. 28283369 10.1016/j.tvjl.2017.02.004

[13] Bergh A, Gómez Álvarez CB, Rhodin M, Gustås P. Head and pelvic vertical displacement in dogs with induced swinging limb lameness: an experimental study. Acta Vet Scand. 2018;60(1):81. 30594234 10.1186/s13028-018-0435-z PMC6311055

[14] Fischer MS, Lilje KE, Lauströer J, Andikfar A. Dogs in motion. 2nd ed. edn: VDH Service. 2014.

[15] Rodriguez O, Regueiro-Purriños M, Figueirinhas P, Gonzalo-Orden JM, Prada I, Vilar JM, Millán L. Rodríguez-Altónaga J: dynamic and postural changes in forelimb amputee dogs: A pilot study. Animals. 2024;14(13):1960. 38998072 10.3390/ani14131960 PMC11240608

[16] Millis DL, Ciuperca IA. Evidence for canine rehabilitation and physical therapy. Veterinary Clinics: Small Anim Pract. 2015;45(1):1–27. 10.1016/j.cvsm.2014.09.001 25432679

[17] Carrier DR, Deban SM, Fischbein T. Locomotor function of the pectoral girdle ‘muscular sling’ in trotting dogs. J Exp Biol. 2006;209(Pt 11):2224–37. 16709923 10.1242/jeb.02236

[18] Cullen KL, Dickey JP, Brown SH, Nykamp SG, Bent LR, Thomason JJ, Moens NM. The magnitude of muscular activation of four canine forelimb muscles in dogs performing two agility-specific tasks. BMC Vet Res. 2016;13:1–13. 10.1186/s12917-017-0985-8 PMC5341356 28270140

[19] Johnson JM, Johnson AL, Pijanowski GJ, Kneller SK, Schaeffer DJ, Eurell JA, Smith CW, Swan KS. Rehabilitation of dogs with surgically treated cranial cruciate ligament-deficient stifles by use of electrical stimulation of muscles. Am J Vet Res. 1997;58(12):1473–8. 9401702

[20] Pelizzari C, Raiser A, Mazzanti A, Salbego F, Festugatto R, Beckmann D, Mori da Cunha MG, Santos R, Serafini G, Marques J, et al. Different times of neuromuscular electrical stimulation medium frequency (kotz) in dogs. Ciencia Rural. 2011;41:1593–9.

[21] GmbH V, Verlag V. Essential facts of physical medicine, rehabilitation and sports medicine in companion animals. VBS Vet. 2019.

[22] Gouveia D, Cardoso A, Carvalho C, Almeida A, Gamboa O, Ferreira A, Martins Â. Approach to small animal neurorehabilitation by locomotor training: an update. Animals. 2022;12:3582. 36552502 10.3390/ani12243582 PMC9774773

[23] Gouveia D, Carvalho C, Cardoso A, Gamboa Ó, Almeida A, Ferreira A, Martins Â. Early locomotor training in tetraplegic Post-Surgical dogs with cervical intervertebral disc disease. Animals. 2022;12(18):2369. 36139228 10.3390/ani12182369 PMC9495086

